# Circulating endothelial cells and other angiogenesis factors in pancreatic carcinoma patients receiving gemcitabine chemotherapy

**DOI:** 10.1186/1471-2407-12-268

**Published:** 2012-06-25

**Authors:** Shunsuke Kondo, Hideki Ueno, Jun Hashimoto, Chigusa Morizane, Fumiaki Koizumi, Takuji Okusaka, Kenji Tamura

**Affiliations:** 1 Hepatobiliary and Pancreatic Oncology Division, National Cancer Center Hospital, Tokyo, Japan; 2 Shien-Lab, National Cancer Center Hospital, Tokyo, Japan; 3 Breast and Medical Oncology Division, National Cancer Center Hospital, Tokyo, Japan

**Keywords:** Pancreatic carcinoma, Circulating endothelial cells, Angiogenesis factors

## Abstract

**Background:**

Pancreatic carcinoma is a significant cause of cancer-related death in developed countries. As the level of circulating endothelial cells (CECs) is known to increase in response to various cancers, we investigated the predictive potential of CEC levels and the association of these levels with the expression of proangiogenic factors in pancreatic carcinoma patients.

**Methods:**

Pancreatic carcinoma patients receiving gemcitabine chemotherapy were prospectively assigned to this study. CEC levels were measured using the CellTracks system, and the plasma levels of several angiogenesis factors were measured using multiplex immunoassay. Associations between clinical outcomes and the levels of these factors were evaluated.

**Results:**

Baseline CEC levels were markedly higher in pancreatic carcinoma patients (n = 37) than in healthy volunteers (n = 53). Moreover, these high CEC levels were associated with decreased overall survival (median, 297 days versus 143 days, *P* < 0.001) and progression-free survival (median, 150 days versus 64 days, *P* = 0.008), as well as with high vascular endothelial growth factor, interleukin (IL)-8, and IL-10 expression in the pancreatic carcinoma patients.

**Conclusions:**

Several chemokines and proangiogenic factors correlate with the release of CECs**,** and the number of CECs detected may be a useful prognostic marker in pancreatic carcinoma patients undergoing gemcitabine chemotherapy.

**Trial registration:**

UMIN000002323

## Background

Pancreatic carcinoma is one of the most lethal tumors and is the fourth leading cause of cancer-related death in developed nations [1]. As pancreatic carcinoma has a high propensity for both local invasion and distant metastasis, surgery is precluded as a treatment for most patients who present with advanced-stage disease. These patients have a median survival of only 6 months and an overall 5-year survival of less than 5%. The prognosis for advanced pancreatic carcinoma patients is therefore extremely poor, and the impact of standard therapy is only modest, despite many advances that have improved the outcome of this disease.

Pancreatic carcinoma is not a grossly vascular tumor; however, it overexpresses multiple mitogenic growth factors that are also angiogenic, such as epidermal growth factor (EGF), hepatocyte growth factor (HGF), fibroblast growth factor (FGF), platelet-derived growth factor B chain (PDGF-BB), and vascular endothelial growth factor (VEGF). Angiogenesis often occurs in response to an imbalance in which proangiogenic factors predominate over antiangiogenic factors. For instance, VEGF expression has been shown to promote tumor growth in pancreatic carcinomas [2]. High VEGF expression is also associated with increased microvessel density [3] and is a predictor of poor outcomes and early tumor recurrence after curative resection [4]. Although agents that target the VEGF signaling pathway have been shown to inhibit tumor growth, metastasis, and angiogenesis [5], treating advanced pancreatic carcinoma patients with axitinib—a selective inhibitor of VEGF receptors 1, 2, and 3—in combination with gemcitabine was not found to improve overall survival in a phase 3 trial [6]. Despite this finding, proangiogenic factors remain an important therapeutic target for the treatment of pancreatic carcinoma.

Circulating endothelial cells (CECs) are mature cells that are not associated with vessel walls but are detached from the endothelium and circulate within peripheral blood. The number of CECs present in the blood has been found to increase in response to cardiovascular disease, vasculitis, infectious disease, and various cancers [7,8]. Indeed, the level of CECs has been recognized as a useful biomarker for vascular damage. It has also been reported that the number of CECs found in non-small cell lung cancer patients treated with carboplatin plus paclitaxel is a promising predictive marker of the clinical efficacy of these drugs [9]. We believe that CEC levels may also be a potential biomarker for pancreatic carcinoma; therefore, we investigated the levels of CECs found in patients with different severities of pancreatic carcinoma, as well as the effects of gemcitabine treatment on CEC levels. Furthermore, the associations between CEC levels and the expression levels of several factors involved in angiogenesis and neovascularization were also examined in this study.

## Methods

### Study approval

This prospective study was approved by the Institutional Review Board of the National Cancer Center, and written informed consent was obtained from all patients. This study is registered with the University Hospital Medical Information Network in Japan (UMIN; number UMIN000002323) and has been completed.

### Patients and blood sample collection

A total of 37 chemotherapy-naïve patients with histologically or cytologically confirmed invasive ductal pancreatic carcinoma were prospectively enrolled in this study between April 2009 and March 2010 and received gemcitabine chemotherapy. Patients with coexisting infections and/or cardiovascular illness were excluded. The detailed history of all the patients was obtained and a physical examination was performed before beginning gemcitabine treatment. Pretreatment baseline laboratory parameters were also assessed for all patients. The baseline tumor status of each patient was evaluated using computed tomography (CT) scans of the chest, abdomen, and pelvis, while peripheral blood sampling was performed both prior to treatment initiation (baseline) and at day 28 ± 7 after starting chemotherapy. A dose of 1000 mg/m^2^ gemcitabine was administered intravenously for 30 min on days 1, 8, and 15 of a 28-day cycle until disease progression, unacceptable toxicity, or patient refusal occurred. The data collected included those pertaining to standard demographics and disease characteristics, the date of initial treatment, the best response to treatment, date of progression, and the date of death or last follow-up. The tumors were evaluated every 6–8 weeks after starting each course of gemcitabine, and best responses were documented according to the Response Evaluation Criteria in Solid Tumors (RECIST).

### CEC enumeration

Blood samples from advanced pancreatic carcinoma patients were drawn into 10 mL CellSave Preservative Tubes (Immunicon Corp. Huntingdon Valley, PA) for CEC enumeration. Samples were obtained both before starting chemotherapy (baseline) and at 28 ± 7 days after starting chemotherapy. Samples were kept at room temperature and processed within 42 h of collection. All of the evaluations were performed without knowledge of the clinical status of the patients. The CellTracks system (Veridex, LLC), which consists of the CellTracks AutoPrep system and the CellSpotter Analyzer system, was used for endothelial cell enumeration. In this system, CECs are defined as CD146^+^/DAPI^+^/CD105-PE^+^/CD45APC^-^ cells. Briefly, CD146^+^ cells were captured immunomagnetically by using ferrofluids coated with CD146 antibodies. The enriched cells were then labeled with the nuclear dye 4 V, 6-diamidino-2-phenylindole (DAPI), CD105 antibodies were conjugated to phycoerythrin (CD105-PE), and the pan-leukocyte antibody CD45 was conjugated to allophycocyanin (CD45-APC). Cells with the DAPI^+^/CD105^+^/CD45^-^ phenotype were enumerated. We evaluated morphological cell viability and excluded dead cells from the cell count. The number of CECs in each sample was determined twice, and the mean value was calculated.

### Antibody suspension bead array system

Peripheral blood was drawn into prechilled tubes containing ethylenediaminetetraacetic acid; was immediately subjected to centrifugation at 1000 g and 4°C for 15 min, plasma was transferred to microtubes and subjected to further centrifugation at 10,000 g and 4°C for 10 min to remove contaminating platelets. Plasma samples were collected from patients before gemcitabine treatment was initiated and were stored at −80°C until they were used for testing. The plasma concentrations of 7 biological markers (interleukin [IL]-6, IL-8, IL-10, PDGF-BB, VEGF, HGF, and SDF-1 alpha) were assayed in a subgroup of patients and control individuals by using the Bio-Plex suspension array system (Bio-Rad, Hercules, CA), which allows the simultaneous identification of cytokines in a 96-well filter plate. In brief, the appropriate cytokine standards and diluted plasma samples were added to a 96-well filter plate and incubated at room temperature for 30 min with antibodies chemically attached to fluorescent-labeled micro beads. After 3 filter washes, premixed detection antibodies were added to each well and incubated for 30 min. After 3 more washes, premixed streptavidin-phycoerythrin was added to each well and incubated for 10 min, followed by 3 more washes. The beads were then resuspended in 125 μL of assay buffer and the reaction mixture was quantified using the Bio-Plex protein array reader. Data were automatically processed and analyzed with Bio-Plex Manager Software 4.1 by using the standard curve obtained using a recombinant cytokine standard.

### Statistical analyses

The Mann–Whitney test was used to compare the distributions of clinical factors and marker concentrations between patients with progressive disease (PD) and those without PD, stages III and IV disease, or recurrence. The survival time (progression-free survival [PFS] and overall survival [OS]) and clinical factors (age, gender, and Eastern Cooperative Oncology Group [ECOG] performance status [PS], and clinical stage of the patients) were examined using the Cox proportional hazards model. The survival curves for PFS and OS were estimated using the Kaplan-Meier method. Kaplan-Meier curves were used only to determine the trends of the associations between the molecules and PFS/OS, as any determination of the optimal cutoff point for the molecules relative to PFS/OS was beyond the scope of the present study. All statistical analyses were performed using IBM SPSS Statistics 18 (IBM Corporation, Somers, NY, USA).

## Results

### Patient characteristics

A total of 37 patients with pancreatic carcinoma were prospectively enrolled in this study. Fourteen of these patients (38%) presented with locally advanced pancreatic carcinoma, 20 patients (54%) presented with metastases, and 3 patients (8%) were enrolled following recurrence after surgery. Twenty-three patients (62%) had ECOG PS0, 10 patients (27%) had ECOG PS1, and 4 patients (11%) had ECOG PS2. Histologically, 14 patients (38%) had poorly differentiated adenocarcinoma, 14 patients (38%) had moderately differentiated adenocarcinoma, 1 patient (2%) had an adenosquamous tumor, and 8 patients (22%) had cytological adenocarcinoma. No patient experienced a complete response to treatment. Four patients (11%) exhibited a partial response (PR) rate to treatment (11%), stable disease (SD) was observed in 22 patients (59%), and PD was observed in 11 patients (30%). Second-line therapy was administered to 20 patients (54%), whereby 18 patients (49%) received S-1 monotherapy and 2 patients (5%) received oxaliplatin and S-1 combination therapy (Table 1).

**Table 1:** Patient characteristics and CEC detection

		**Mean CEC level 166 cells/4 mL**	**Range (2–1195 cells/4 mL)**	**Total**	***P***^***a***^
		**≥ 166 cells/4 mL**	**<166 cells/4 mL**		
		**CEC**^**high**^	**CEC**^**low**^		
		12	25	37	
Age	Over 70	8	10	18 (49%)	0.17
	Below 70	4	15	19 (51%)	
Sex	Male	7	17	24 (65%)	0.72
	Female	5	8	13 (35%)	
Stage	III	3	11	14 (38%)	0.59
	IV	8	12	20 (54%)	
	Recurrence	1	2	3 (8%)	
ECOG PS	0	5	18	23 (62%)	0.09
	1	6	4	10 (27%)	
	2	1	3	4 (11%)	
Pancreatic tumor location	Head	5	12	17 (46%)	>0.9
	Body	5	9	14 (38%)	
	Tail	2	4	6 (16%)	
CA19-9 (U/mL)	≥10,000	3	5	8 (22%)	>0.9
	< 10,000	9	20	29 (78%)	
CRP (mg/dL)	≥1.0	7	3	10 (27%)	<0.01
	<1.0	5	22	27 (73%)	
Histology	Poorly differentiated	5	9	14 (38%)	0.62
	Moderately differentiated	4	10	14 (38%)	
	Adenosquamous	1	0	1 (2%)	
	N.E (cytology only)	2	6	8 (22%)	
Tumor response	Partial response	2	2	4 (11%)	<0.05
	Stable disease	4	18	22 (59%)	
	Progressive disease	6	5	11 (30%)	
Second line therapy	S-1	6	12	18 (49%)	1
	Oxaliplatin + S-1	0	2	2 (5%)	
	No	6	11	17 (46%)	

^*a*^*P* values were calculated for each variable using Fisher’s exact test.

Abbreviations: CEC = circulating endothelial cell; ECOG = Eastern Cooperative Oncology Group; CA19-9 = carbohydrate antigen 19–9; CRP = C-reactive protein.

### Baseline levels of CECs and angiogenic factors

**Figure 1:**
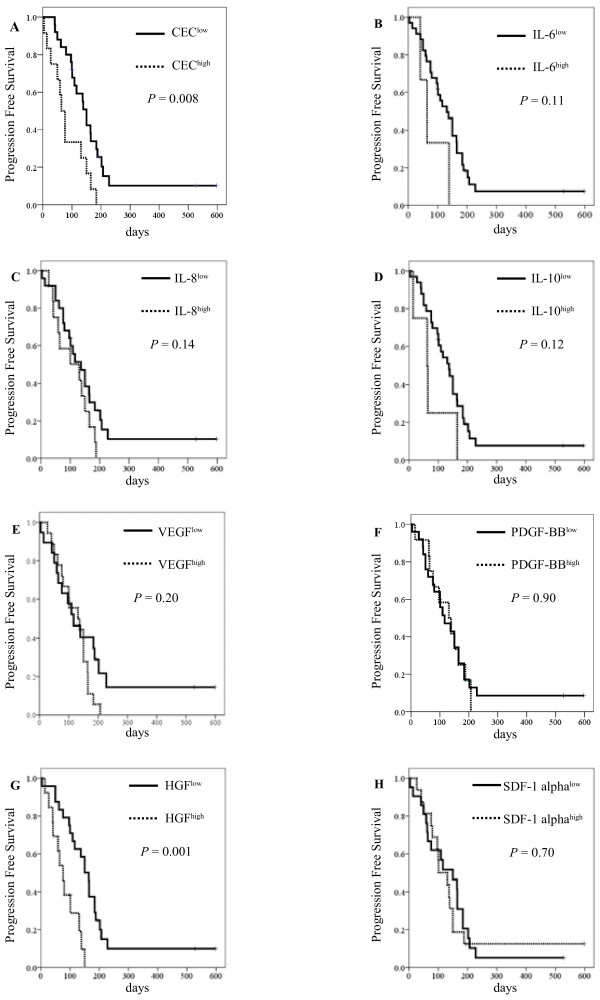
**Kaplan-Meier curves for (A) progression-free survival with CEC counts, (B) progression-free survival with IL-6 levels, (C) progression-free survival with IL-8 levels, (D) progression-free survival with IL-10 levels, (E) progression-free survival with VEGF levels, (F) progression-free survival with PDGF-BB levels, (G) progression-free survival with HGF levels, and (H) progression-free survival with SDF-1 alpha levels.** The cut-off points for the angiogenic factors were determined to be equal to or greater than these mean levels.

**Figure 2:**
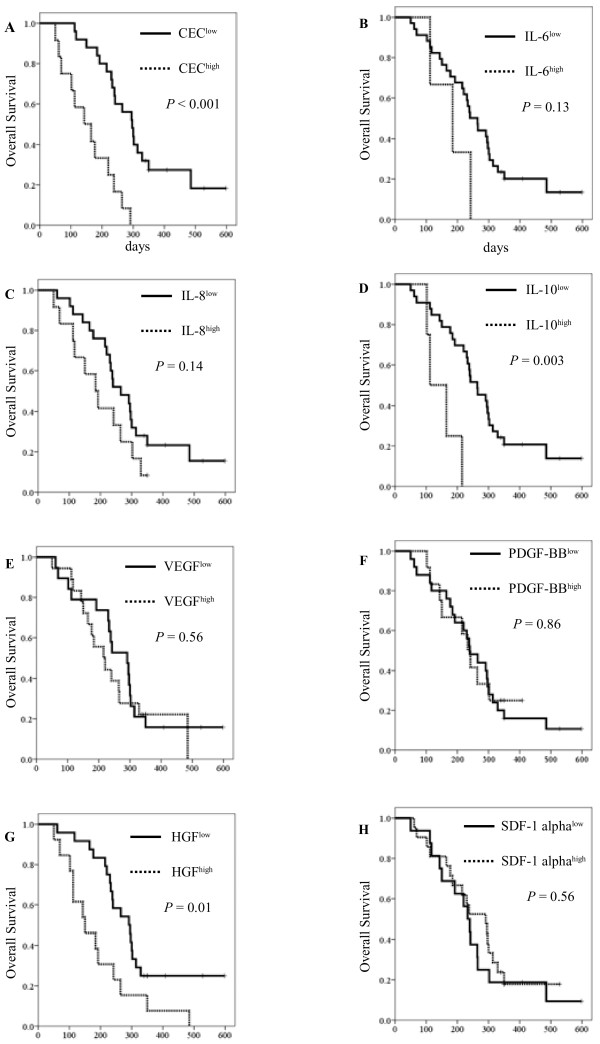
**Kaplan-Meier curves for (A) overall survival with CEC counts, (B) overall survival with IL-6 levels, (C) overall survival with IL-8 levels, (D) overall survival with IL-10 levels, (E) overall survival with VEGF levels, (F) overall survival with PDGF-BB levels, (G) overall survival with HGF levels, and (H) overall survival with SDF-1 alpha levels.** The cut-off points for the angiogenic factors were determined to be equal to or greater than these mean levels.

The mean CEC level found in the pancreatic carcinoma patients was 166 cells/4 mL (range: 2–1195 cells/4 mL) while the median CEC level was 66 cells/4 mL. These CEC levels were higher than those of randomly-selected healthy volunteers (*P* < 0.01), as previously reported (n = 53, mean ± SD = 46.2 ± 86.3 cells/4 mL) [9]. In this study, the cut-off point of CEC^high^ was determined to be equal to or greater than 166 cells/4 mL while that of CEC^low^ was lower than 166 cells/4 mL. CEC^high^ was significantly associated with high levels of C-reactive protein (CRP) (over 1.0 mg/dL; *P* < 0.01). The median PFS was 64 days (95% confidence interval [CI], 45–83) in the CEC^high^ group, while that in the CEC^low^ group was 150 days (95% CI, 130–170; log-rank test; *P* = 0.008; Figure 1A). The median OS was 143 days (95% CI, 53–233) in the CEC^high^ group and 297 days (95% CI, 240–354) in the CEC^low^ group (log-rank test; *P* < 0.001; Figure 2A). Univariate analysis of CEC levels and clinical factors for OS was performed using the Cox proportional hazard model. The hazard ratio (HR) for CEC levels (CEC^high^ versus CEC^low^) was 5.18 (95% CI, 2.23–12.03; *P* < 0.001).

The mean levels of IL-6, IL-8, IL-10, PDGF-BB, VEGF, HGF, and SDF-1 alpha were found to be 19.3 pg/mL, 11.3 pg/mL, 7.82 pg/mL, 1127.5 pg/mL, 44.1 pg/mL, 471.3 pg/mL, and 110.6 pg/mL, respectively. The cut-off points for the angiogenic factors were determined to be equal to or greater than these mean levels, and the median PFS in HGF^low^ was longer than the HGF^high^ group (*P* = 0.001; Figure 1 G). However, other factors were not found to have statistical significance with regard to PFS. The median OS was longer in the case of IL-10 (112 days [95% CI, 50–173] in IL-10^high^ vs. 264 days [95% CI, 204–324] IL-10^low^, log-rank test: *P* = 0.003; Figure 2d) and HGF (150 days [95% CI, 65–234] in HGF^high^ vs. 291 days [95% CI, 223–359] in HGF^low^, log-rank test: *P* = 0.01; Figure 2 G).

Among the clinical factors that were examined in this study, a poor PS (PS 1 and 2), advanced stage (stage IV and recurrence), and high levels of IL-10, HGF, and CRP were significantly correlated with poor OS in univariate cox analysis, with HRs of 2.72 (95% CI, 1.29–5.70; *P* = 0.008), 2.21 (95% CI, 1.03–4.71; *P* = 0.04), 5.05 (95% CI, 1.55–16.39; *P* = 0.007), 2.52 (95% CI, 1.22–5.21; *P* = 0.01), and 2.49 (95% CI, 1.14–5.42; *P* = 0.02), respectively. In a multivariate Cox analysis model that included clinical stage, PS, CRP levels, CEC levels, IL-10 levels, and HGF levels, the number of CECs detected remained statistically stable at 0.05. The resulting HRs were 2.04 (95% CI, 0.78–5.35; *P* = 0.15), 2.58 (95% CI, 0.98–6.76; *P* > 0.05), 2.04 (95% CI, 0.62–6.76; *P* = 0.24), 5.14 (95% CI, 1.83–14.45, *P* = 0.002), 5.26 (95% CI, 1.26–22.22; *P* = 0.02) and 1.34 (95% CI, 0.46–3.91; *P* = 0.59), respectively (Table 2).

**Table 2:** Univariate and multivariate Cox analyses of prognosis

**Univariate analysis**	**HR**	**95% CI**	***P***
Age: Over 70 vs. Below 70	0.52	0.25–1.13	0.1
Sex: Male vs. Female	1.00	0.48–2.08	0.99
Stage: IV + Recurrence vs. III	2.21	1.03–4.71	0.04
ECOG PS: 2 + 1 vs. 0	2.72	1.29–5.70	0.008
Pancreatic tumor location: Head vs. Others	0.94	0.46–1.90	0.86
CA19-9 (cut-off: 10,000 U/mL): CA19-9^high^ vs. CA19-9^low^	1.77	0.75–4.15	0.19
CRP level (cut-off: 1.0 mg/dL): CRP^high^ vs. CRP^low^	2.49	1.14–5.42	0.02
Histology: Poorly differentiated vs. Others	1.09	0.52–2.27	0.82
Second line therapy: Yes vs. No	0.61	0.30–1.24	0.17
CEC level (cut-off: 166 cells/4 mL): CEC^high^ vs. CEC^low^	5.18	2.23–12.03	<0.001
IL-6 (cut-off: 19.3 pg/mL): IL-6^high^ vs. IL-6^low^	2.52	0.73–8.64	0.14
IL-8 (cut-off: 11.3 pg/mL): IL-8^high^ vs. IL-8^low^	1.74	0.82–3.67	0.15
IL-10 (cut-off: 7.82 pg/mL): IL-10^high^ vs. IL-10^low^	5.05	1.55–16.39	0.007
VEGF (cut-off: 44.1 pg/mL): VEGF^high^ vs. VEGF^low^	1.22	0.60–2.47	0.59
PDGF-BB (cut-off: 1127.5 pg/mL): PDGF-BB^high^ vs. PDGF-BB^low^	0.93	0.43–2.04	0.86
HGF (cut-off: 471.3 pg/mL): HGF^high^ vs. HGF^low^	2.52	1.22–5.21	0.01
SDF-1 alpha (cut-off: 110.6 pg/mL): SDF-1 alpha^high^ vs. SDF-1 alpha^low^	1.23	0.60–2.53	0.56
**Multivariate analysis**	**HR**	**95% CI**	***P***
Stage: IV + Recurrence vs. III	2.04	0.78–5.35	0.15
ECOG PS: 2 + 1 vs. 0	2.58	0.98–6.76	>0.05
CRP level (cut-off: 1.0 mg/dL): CRP^high^ vs. CRP^low^	2.04	0.62–6.76	0.24
CEC level (cut-off: 166 cells/4 mL): CEC^high^ vs. CEC^low^	5.14	1.83–14.45	0.002
IL-10 (cut-off: 7.82 pg/mL): IL-10^high^ vs. IL-10^low^	5.26	1.26–22.22	0.02
HGF (cut-off: 471.3 pg/mL): HGF^high^ vs. HGF^low^	1.34	0.46–3.91	0.59

Abbreviations: HR = hazard ratio; CI = confidence interval; ECOG PS = Eastern Cooperative Oncology Group performance status; CEC = circulating endothelial cells; IL = interleukin; PDGF-BB = platelet-derived growth factor-B chain; VEGF = vascular endothelial growth factor; HGF = hepatocyte growth factor; CA19-9 = carbohydrate antigen 19–9; CRP = C-reactive protein; CEA = carcinoembryonic antigen.

### Changes in CEC number during treatment

**Figure 3:**
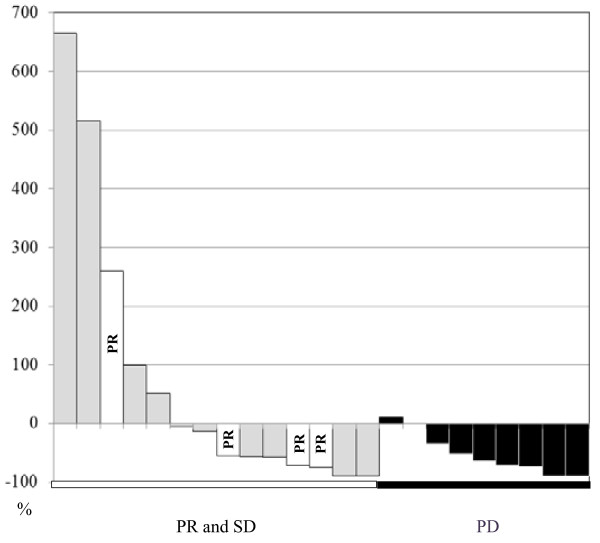
Waterfall plot showing the changes in CEC counts and tumor response in patients without progressive disease (PD) (those with partial response [PR] or stable disease [SD]) and patients with PD, after 28 ± 7 days of gemcitabine treatment.

The number of CECs was analyzed in 22 of the 37 patients at 28 ± 7 days after the start of gemcitabine therapy. The mean number of CECs detected in these patients after 28 ± 7 days was 133 cells/4 mL (range: 15–664 cells/4 mL), while the median number of CECs was 68 cells/4 mL. The absolute counts of CECs did not change significantly between day 1 and day 28 ± 7 of treatment (Mann–Whitney test, *P* = 0.11). Furthermore, a change in CEC counts from baseline to after 28 ± 7 days of treatment was not statistically associated with tumor response (Mann–Whitney test, *P* > 0.05, Figure 3).

### Association between CEC number and blood angiogenic factors

The numbers of CECs were compared between non-PD (PR and SD, n = 26) and PD patients (n = 11) for all markers. The baseline levels of CEC (*P* = 0.03), IL-6 (*P* < 0.01), and IL-10 (*P* = 0.03) were found to be significantly higher among patients with PD than among those with PR or SD. The blood concentrations of HGF (*P* < 0.001), IL-6 (*P* < 0.01), and IL-8 (*P* < 0.001) were also significantly higher among patients with clinical stage IV disease and recurrence than among those with stage III disease. When the association between CEC number and the expression of other angiogenic factors was examined, the number of CECs was found to correlate positively with the levels of VEGF (r = 0.34, *P* = 0.04), HGF (r = 0.37, *P* = 0.02), IL-8 (r = 0.38, *P* = 0.02), and IL-10 (r = 0.45, *P* = 0.006), suggesting that the number of CECs is related to the expression of these markers (Table 3).

**Table 3:** Association between CECs and other factors

	**Mean ± SD**	**Spearman’s rank correlation coefficient**	***P***
CEC (cells/4 mL)	166.2 ± 228.9	1	-
IL-6 (pg/mL)	19.3 ± 52.4	0.17	0.30
IL-8 (pg/mL)	11.3 ± 10.1	0.38	0.02
IL-10 (pg/mL)	7.82 ± 26.9	0.45	0.006
VEGF (pg/mL)	44.1 ± 38.8	0.34	0.04
PDGF-BB (pg/mL)	1,127.5 ± 941.5	0.24	0.16
HGF (pg/mL)	471.3 ± 249.0	0.37	0.02
SDF-1alpha (pg/mL)	110.6 ± 43.7	0.15	0.37
CRP (mg/dL)	1.9 ± 3.9	0.31	0.06
CA19-9 (U/mL)	18,229.1 ± 55,377.8	0.11	0.50
CEA (ng/mL)	18.3 ± 51.0	0.03	0.88

Abbreviations: CEC = Circulating endothelial cell; IL = interleukin; PDGF-BB = platelet-derived growth factor-B chain; VEGF = vascular endothelial growth factor; HGF = hepatocyte growth factor; CA19-9 = carbohydrate antigen 19–9; CRP = C-reactive protein; CEA = carcinoembryonic antigen.

## Discussions

In most cases, CECs are apoptotic or necrotic cells that are released into circulation as a byproduct of vascular turnover. In some cancer patients, the level of CECs is significantly higher than that of healthy individuals, and this increased level has been identified as a surrogate marker of angiogenesis and anti-angiogenic drug activity [10,11]. The present study has shown that baseline CEC levels are markedly higher among pancreatic carcinoma patients than in healthy individuals. Our results also support the hypothesis that CEC levels are associated with clinical outcome in pancreatic carcinoma patients undergoing gemcitabine chemotherapy, and may be a prognostic factor for this disease. A previous study found that the baseline level of CECs, identified as CD45^-^CD31^+^CD34^+^ by flow cytometry, was inversely associated with OS in patients who had gemcitabine-refractory metastatic pancreatic carcinoma and were treated with bevacizumab plus erlotinib [12]. CEC (CD45^-^CD31^+^CD146^+^) detection by flow cytometry requires careful discrimination between blood cell populations with overlapping phenotypes showing hallmarks of T cells (CD45^-^CD31^-^CD146^+^) and platelets (CD45^-^CD31^high^CD146^-^). These cells populations show distinct regulation during cancer therapy, and their concomitant analysis may offer extended prognostic and predictive information [13].

Our study also found the baseline level of CECs, as well as the levels of HGF, IL-6, and IL-10, which are associated with gemcitabine resistance or stemness, to be significantly higher among PD patients. Univariate Cox model analysis further demonstrated that PS, clinical stage, CRP levels, and CEC levels are all associated with the survival of pancreatic carcinoma patients, while multivariate Cox analysis showed that CEC and IL-10 levels are strongly associated with survival.

The number of CECs detectable in individuals has previously been found to be associated with the plasma levels of VCAM-1 and VEGF in cancer patients [14][15]. Our findings further show that, in addition to VEGF, CEC levels are strongly associated with the expression levels of IL-8, IL-10, and HGF in pancreatic carcinoma patients. These molecules, among others, play important roles in tumor biology and have been implicated in several cellular phenotypes. Chemokines, including IL-8 and IL-10, are small peptides involved in controlling cell migration, particularly in leukocytes, during inflammation and the immune response. Chemokines are also important in tumor biology as they influence tumor growth, invasion, metastasis, and angiogenesis. For instance, VEGF, HGF and IL-8 significantly stimulate the proliferation, migration, and invasion of cancer cells. CEC are shed from vessels and this process may be amplified by an aberrant vascular turnover/remodeling associated with high local levels of VEGF required for CEC survival [16]. The chemokine SDF-1 has likewise been found to enhance the production of IL-8 by pancreatic cells in a paracrine manner [17]. Although our results did not indicate that SDF-1 levels were associated with CEC or IL-8 levels in the pancreatic cancer patients examined, it is likely that several of the proangiogenic factors examined in this study interact with each other to promote vascular turnover and remodeling, thereby leading to a higher number of CECs in the peripheral blood of cancer patients.

Drugs targeting angiogenesis, such as those that inhibit the VEGF pathway, have had a major impact in the treatment of many types of cancer. The VEGF pathway is also an independent prognostic factor for patient survival in pancreatic carcinoma. Although preclinical models have suggested that VEGF-VEGF receptor inhibitors would be effective in the treatment of pancreatic carcinoma, patients who received bevacizumab and axitinib therapy in addition to gemcitabine have not shown a survival advantage when compared to those treated with gemcitabine alone [6,18]. These results add to the increasing evidence that suggests that targeting VEGF signaling is an ineffective strategy in the treatment of pancreatic carcinoma. However, many antiangiogenic therapies modulate the expression levels of proangiogenic factors [19], and many factors are associated with tumor angiogenesis. Therefore, there are a variety of potential therapeutic targets that may be exploited in order to target angiogenesis, potentially including those examined in this study.

In advanced non-small cell lung cancer (NSCLC), patients with higher baseline CEC counts have PR/SD and longer PFS. It has also previously been reported that the elevated CEC numbers exhibited in NSCLC patients decrease following treatment with carboplatin in combination with paclitaxel [9]. Paclitaxel and docetaxel are categorized as mitotic spindle agents with potent antiangiogenic properties [20-22]. Therefore, it seems that the baseline CEC count is a promising predictor of clinical response to the carboplatin plus paclitaxel regimen, as well as of survival. However, although several other clinical studies that have examined CECs have also found chemotherapy to be associated with either an increase or decrease in CEC number [23,24], no association was detected between gemcitabine treatment and CEC number in the pancreatic carcinoma patients in our study. Although gemcitabine has anti-angiogenic properties, higher baseline CEC levels were associated with PD in pancreatic carcinoma patients receiving gemcitabine therapy, and patients with high CEC counts exhibited poor clinical condition. It is therefore likely that the tumor type, anti-cancer drugs being administered, and the amount of time between the start of treatment and the time when CEC counts are obtained influence the number of CECs detected in cancer patients after treatment. In this study, we measured CEC levels before starting chemotherapy and at 28 ± 7 days after starting chemotherapy, the time of sampling might influence the changes of CEC level. Moreover, the diversity in literature regarding CEC up-or down-regulation during cancer therapy and the associated prognostic and predictive evidence might in part be explained by a differential focus on or by the lack of discrimination between these cell populations [13]**.**

## Conclusions

Although the number of patients examined in this study was small, and patients were recruited prospectively, this study, along with others, has shown the clinical importance of CEC number as a prognostic factor in advanced pancreatic carcinoma treated with gemcitabine chemotherapy, whereby high CEC counts are associated with poor prognosis. This study also found that elevated CEC counts are associated with the high expression levels of several chemokines and proangiogenic factors involved in the regulation of tumor immunological and angiogenic factors. Although this correlation between blood parameters is not proof of a causal relationship, these factors may provide viable therapeutic targets for the treatment of pancreatic carcinoma in the future. Further studies in a larger population will be required to confirm our findings.

## Abbreviations

CEC, circulating endothelial cell; ECOG , Eastern Cooperative Oncology Group; CA19-9, Carbohydrate antigen 19–9; CRP, C-reactive protein; IL, Interleukin; PDGF-BB, Platelet-derived growth factor-B chain; VEGF, Vascular endothelial growth factor; HGF, Hepatocyte growth factor; PD, Progressive disease; PR, Partial response; HR, Hazard ratio; CI, confidence interval; SD, Stable disease.

## Competing interests

The authors declare that they have no competing interests.

## Authors’ contributions

SK and KT designed and participated in all stages of the study. SK and JH performed most of the experiments. FK and CM participated in CEC analysis, as well as the statistical analyses and discussion of the results. HU and TO recruited the patients, collected the tumor biopsy samples, and helped to draft the manuscript. All authors read and approved the final manuscript.

## Pre-publication history

The pre-publication history for this paper can be accessed here:

http://www.biomedcentral.com/1471-2407/12/268/prepub
